# First Synthesis of Shimobashiric Acid C With Acetonide as the Catechol Protecting Group

**DOI:** 10.1002/cbdv.71738

**Published:** 2026-09-30

**Authors:** Ruwen Li, Zhe Zhang, Pengshu Xu, Lin Zhou, Lei Chen, Zhongqiang Liu

**Affiliations:** ^1^ School of Pharmaceutical Sciences Hainan University Haikou Hainan Province P.R. China

**Keywords:** acetonide protection, *D*‐Danshensu, photodimerization, shimobashiric acid C, α‐truxillic acid

## Abstract

Shimobashiric acid C (SBA‐C) shows excellent hyaluronidase inhibition but its natural isolation yield is only 0.0092 %. We report its first synthesis via a convergent seven‐step route from caffeic and rosmarinic acids in 35% overall yield. Direct protection of *D*‐Danshensu (*D*‐DSS) with 2,2‐dimethoxypropane gave an isochroman; the desired acetonide‐protected *D*‐DSS building block was instead prepared from methyl rosmarinate via bis‐acetonide formation followed by methanolysis. Acetonide‐protected caffeic acid was synthesized and crystallized in a *P*‐1 lattice, where linear hydrogen‐bonded caffeic acid pairs pack with adjacent alkenyl bonds aligned at 3.919 Å for head‐to‐tail solid‐state [2+2] photodimerization. Irradiation at 365 nm gave the acetonide‐protected *α*‐truxillic acid building block. The two blocks were condensed via Steglich esterification to give acetonide‐protected SBA‐C methyl ester. Selective deprotection of the acetonide groups was achieved with 55% trifluoroacetic acid in the presence of trace water in nearly quantitative yields. This mild deprotection caused no noticeable side reactions and may be applied to synthesis of other *D*‐DSS‐containing phenolic acids, such as salvianolic acids.

## Graphical abstract

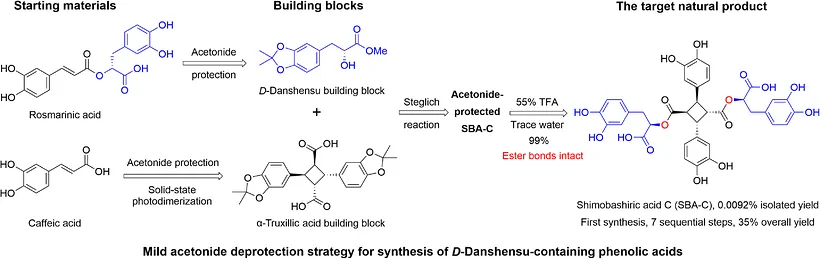

## 1 Introduction

Hyaluronic acid (HA) is a fundamental component of the extracellular matrix in connective tissues [1, 2] and its turnover is regulated by hyaluronidases, the overexpression of which is closely associated with pathological processes such as tumor metastasis and autoimmune disorders [3]. Naturally occurring hyaluronidase inhibitors have thus attracted considerable interest as potential drug candidates. Shimobashiric acid C (SBA‐C, Figure 1), first isolated from *Keiskea japonic*a with a very low yield of 0.0092 wt%, stands out for its excellent inhibitory activity [4, 5]. SBA‐C is a phenolic acid consisting of two *D*‐Danshensu (*D*‐3,4‐dihydroxyphenyllactic acid, *D*‐DSS) residues linked through ester bonds to a *trans*‐cyclobutane diacid core, a motif found in *α*‐truxillic acids [6].

**FIGURE 1:**
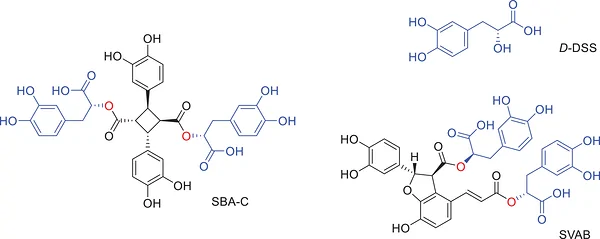
The structures of SBA‐C, *D*‐DSS, and SVAB.

The *D*‐DSS moiety is also found in many other naturally occurring bioactive compounds, such as sagerinic acid, rabdosiin [5], globoidnan B [7], lithospermic acid, and salvianolic acid A, B (SVAB), C, and D, most of which have been widely studied for cardiovascular diseases (CVDs) [8, 9]. The synthesis of these compounds is often hampered by low yields during the removal of catechol protecting groups (e.g., methyl ethers), likely due to significant unfavorable side reactions under harsh deprotection conditions (e.g., with BBr_3_) [10, 11, 12]. In contrast, acetonide, another catechol protecting group, can be removed rapidly, completely, and cleanly under mild conditions using trifluoroacetic acid (TFA), as demonstrated in the synthesis of peptides bearing *L*‐3,4‐dihydroxyphenylalanine (*L*‐DOPA) [13, 14].

The intricate cyclic frameworks of natural products are central to their biological activities [15]. Cycloaddition reactions, such as [5+2], [4+2], and [2+2], along with the Pictet–Spengler reactions, are key to building these diverse structures [16, 17]. For instance, UV‐induced solid‐state [2+2] photodimerization is commonly employed to construct the specific (1*α*, 2*α*, 3*β*, 4*β*) cyclobutane frameworks of *α*‐truxillic acids, often yielding optically pure stereoisomers [18]. However, this requires engineering specific crystal forms in which the two reactive alkenyl bonds are arranged in a head‐to‐tail manner, with an inter‐bond distance falling within the range of 3.5–4.2 Å, as demanded by the Schmidt's rule [19].

To provide accessible and affordable SBA‐C for hyaluronidase inhibition applications and other bioactivity studies, and to explore alternative strategies for the synthesis of *D*‐DSS‐containing phenolic acids, a convergent synthetic procedure was developed using acetonide as the catechol protecting group and achieved the first synthesis of the natural product SBA‐C and its methyl ester.

## 2 Results and Discussion

### 2.1 Retrosynthetic Analysis

Based on the retrosynthetic principles (Scheme 1), SBA‐C was disassembled into two building blocks: the acetonide‐protected *D*‐DSS methyl ester (**1**) and the acetonide‐protected *α*‐truxillic acid (**2**). The synthesis of each block was designed with a comparable step count to minimize the cumulative product loss arising from long linear sequences [20]. The two blocks were then convergently assembled via Steglich esterification, followed by sequential deprotection, to afford the target product.

**SCHEME 1:**
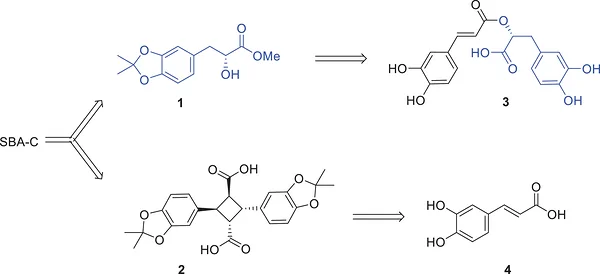
Retrosynthetic analysis of SBA‐C.

The acid‐catalyzed direct protection of *D*‐DSS with acetone or 2,2‐dimethoxypropane (DMP) can be complicated by the oxa‐Pictet–Spengler reaction, which affords isochroman—a thermodynamically more favorable product due to the formation of both an ether and a carbon–carbon single bond [21, 22]. To test this hypothesis (Scheme 2), a crude *D*‐DSS sample (obtained from hydrolysis of SVAB) was first methylated with SOCl_2_/MeOH, and then, without purification, subjected to acetonide cyclization by refluxing with *p*‐toluenesulfonic acid (TsOH) in a mixture of DMP, acetone, and benzene (PhH). From the reaction mixture, only an isochroman product (**5**) was isolated; the desired *D*‐DSS block **1** was not detected. This outcome is consistent with the observations of Messersmith and Maleczka on the acetonide protection of *L*‐DOPA [23, 25]. The test indicated that pre‐protection of the α‐hydroxyl group of *D*‐DSS is necessary to achieve selective acetonide formation on the catechol moiety.

**SCHEME 2:**
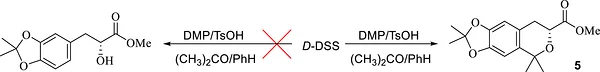
The acetonide product and isochroman product of *D*‐DSS.

Given the high cost of *D*‑DSS, rosmarinic acid (**3**) represents a more economical starting material, as it is a by‑product of rosemary essential oil. Moreover, the α‑hydroxyl group in rosmarinic acid is already masked by a caffeoyl moiety—albeit not a conventional protecting group—thus eliminating the need for an additional pre‐protection step. The *α*‑Truxillic acid block **2** can be constructed from *trans*‐caffeic acid (**4**) via solid‑state [2+2] photodimerization.

### 2.2 Synthesis of the *D*‐DSS Building Block

Like many polyphenolic acids, rosmarinic acid **3** is soluble in water but not in benzene (Scheme 3). To improve its solubility in nonpolar solvents, the polar carboxyl group of **3** was masked as a methyl ester using MeOH and DMP in the presence of chlorotrimethylsilane (Me_3_SiCl). Subsequently, acetonide protection of methyl rosmarinate (**6**) was performed following a procedure reported by Liu and co‑workers [26]. Briefly, ester **6** was dissolved in acetone, mixed with DMP and benzene, and then refluxed in the presence of TsOH. A Soxhlet extractor was equipped with the thimble filled with anhydrous CaCl_2_ to absorb byproducts, thereby shifting the reaction equilibrium. The reaction progress was monitored by reversed‑phase high‑performance liquid chromatography (RP‑HPLC). The results showed that the mono‐acetonide protected methyl rosmarinate (**7**) was initially formed, isolated in 80% yield. Upon further reaction with excess DMP, intermediate **7** was fully consumed, and the bis‐acetonide protected methyl rosmarinate (**8**) was obtained as the main product in 83% yield. This demonstrated the high selectivity of acetonide protection: the catechol of the *D*‐DSS residue is more reactive than the electron‐poor catechol of the caffeoyl moiety, which is deactivated by the conjugated acrylate group.

**SCHEME 3:**
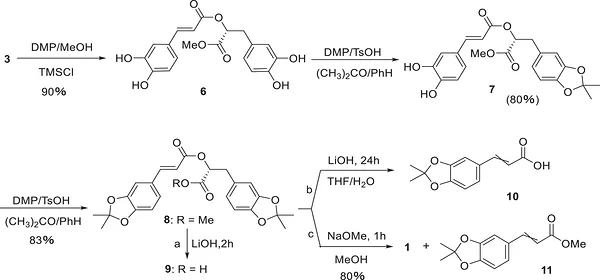
Synthesis of the acetonide‐protected *D*‐DSS methyl ester.

Selective hydrolysis of the secondary ester bond of **8** in the presence of a primary methyl ester proved challenging. At room temperature, two equivalents of LiOH in tetrahydrofuran (THF)/H_2_O preferentially cleaved the methyl ester bond within 2 h to afford acid **9**. However, excess LiOH and prolonged reaction times led to cleavage of both ester bonds and promoted elimination of the released α‑hydroxyl group, giving a mixture of *trans*‐ and *cis*‐caffeic acids (**10**). Ultimately, alcoholysis of **8** with NaOMe in anhydrous MeOH successfully afforded the target acetonide **1**, along with a mixture of *trans‐* and *cis*‐methyl caffeate (**11**). The *cis* isomer was produced in small amount, arising from α‐hydroxyl elimination, whereas the *trans* isomer was the major product, derived from both the caffeoyl protecting group and the same elimination process, as confirmed by NMR analysis. Furthermore, prolonged exposure to methanolysis significantly reduced the yield of block **1** and increased that of compound **11**, as revealed by RP‐HPLC monitoring.

Since the acetonide product and the Pictet–Spengler product of *L*‑DOPA were quite easily confusable [23, 24], the characteristic features of *D*‐DSS block **1** and the oxa‑Pictet–Spengler product **5** are compared and presented in Table 1. At room temperature, both **1** and **5** gave negative results (yellow background) with FeCl_3_ color test on silica thin‐layer chromatography (TLC) plates, indicating full protection of the catechol groups. Upon heating at 105°C for 5 min, both developed positive results (black dots), indicating the presence of an acid‐labile protecting group—in this case, the acetonide group [27]. Although both compounds show the quaternary carbon of the acetonide around δ 118 ppm in the ^1^ ^3^C NMR spectra, striking differences are observed in the DEPT‐135 spectra: block **1** displays three aromatic tertiary carbons in the high chemical shift region (*δ* > 100 ppm), while product **5** exhibits only two, one fewer due to formation of an additional carbon–carbon single bond. Block **1** has no aliphatic quaternary carbon and hence no peak below 100 ppm misses in the DEPT‐135 spectrum. In contrast, product **5** has one at 76.74 ppm, and that peak indeed disappears. The ^1^H NMR spectra are also informative: block **1** shows three aromatic protons by integration whereas product **5** displays only two uncoupled singlets. Unlike *L*‑DOPA, *D*‑DSS can undergo both oxa‑Pictet–Spengler reaction and acetonide cyclization simultaneously, and product **5** exhibits a molecular weight increase consistent with the incorporation of two isopropylidene groups.

**TABLE 1:** Characteristic features of the *D*‐DSS building block **1** and the isochroman product **5**

Properties	*D*‐DSS building block 1	Isochroman product 5
FeCl_3_ test on silica TLC plate, 25°C	negative	negative
FeCl_3_ test on silica TLC plate, 105°C	positive	positive
^13^C NMR C of the acetonide δ ppm	118.71	117.93
DEPT‐135 δ > 100 ppm, CH	122.93, 110.57, 108.73	108.03, 105.10
DEPT‐135 δ < 100 ppm, C (missing)	/	76.74
DEPT‐135 δ < 100 ppm, CH_3_	52.34, 25.90	52.33, 31.39, 28.43, 25.94
^1^H NMR *δ* > 6 ppm, Ar‐H	6.63(d, 1H), 6.61‐6.60(m, 2H)	6.45(s, 1H), 6.43(s, 1H)
HRMS *m*/*z* found	[M+Na]^+^ 275.0885	[M+Na]^+^ 315.1201
Mw increase from the methyl ester	40	80

### 2.3 Synthesis of the α‐Truxillic Acid Building Block

The *α*‐Truxillic acid building block **2** was synthesized from caffeic acid **4** in four steps (Scheme 4). First, to enhance solubility in nonpolar solvents, acid **4** was esterified to methyl caffeate (**12**) using SOCl_2_/MeOH. Subsequently, acetonide protection of **12** was introduced under conditions similar to those for ester **6**, affording **13** in 88% yield. Intermediate **13** was obtained as a sticky residue at room temperature and could not undergo solid‐state photodimerization. Therefore, **13** was hydrolyzed with LiOH in THF/H_2_O to provide the carboxylic acid (**14**) as a crystalline powder.

**SCHEME 4:**
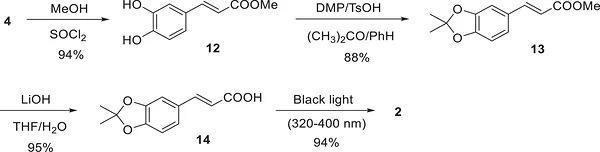
Synthetic route to *α*‐truxillic acid **2**.

Single crystals of **14** were subjected to x‐ray diffraction analysis. Two molecules of **14** form a head‐to‐head linear pair through their carboxyl groups, with an O1···O2 distance of 2.624 Å and an O1···H1···O2 angle of 174.19°, indicating the formation of strong hydrogen bonds (Figure 2). These hydrogen‐bonded pairs of **14** pack into the triclinic space group *P* ‐1 with a longitudinal offset of approximately 5.2 Å. This demonstrates that strong hydrogen bonding drives small molecules into molecular pairs, thereby promoting crystalline solid formation. The distance between the stacking planes is about 3.4 Å at the acryloyl moiety and increases to approximately 4.9 Å (measured from C10 to C4 across the planes) at the benzodioxole moiety, owing to steric effects of the acetonide methyl groups. The olefinic bond of the bottom‑left (BL) molecule is parallel to that of the top‑left (TL) molecule at a distance of 6.623 Å, and is anti‑parallel to that of the top‑right (TR) molecule at a distance of 3.919 Å. According to Schmidt's rule, compound **14** is expected to dimerize in a head‐to‐tail manner.

**FIGURE 2:**
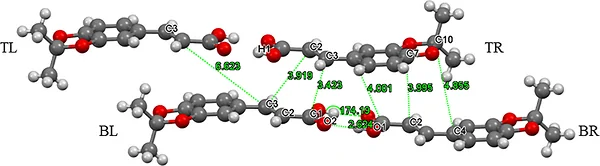
Hydrogen‐bonded pairs of acid **14** and their packing arrangement.

Photodimerization was initially carried out by irradiating a suspension of **14** in 5 vol% EtOH in water with a black‑light lamp (320–400 nm). After several hours, a new peak appeared on the RP‐HPLC chromatogram approximately 0.8 min after that of the reactant **14**, indicating the formation of a new compound, likely a dimer. Ethanol served as a wetting agent to facilitate dispersion of **14** in water; in its absence, photodimerization progressed slowly due to inadequate mixing. Higher percentages of EtOH significantly reduced the conversion rates because photodimerization occurred only in the solid state; the additional **14** that was brought into solution by ethanol did not dimerize. A large‑scale (gram‑level) synthetic protocol was accordingly developed by continuously rotating a Pyrex glass flask over an LED UV lamp (365 nm). Under these conditions, ethanol was unnecessary, as mechanical rotation ensured more effective mixing and UV light exposure.

Single crystals of block **2** were subjected to x‐ray diffraction analysis. The structure confirmed the expected *α*‐truxillic acid‐type relative stereochemistry of the cyclobutane core (Figure 3), with the two bulky aromatic substituents positioned on opposite sides of the ring. Far from being innocent bystanders, protecting groups actively modulate the molecular packing arrangement, thereby directing photodimerization toward distinctly different products: the methylene acetal favors *β*‐truxinic acids [28, 29], whereas the acetonide leads to *α*‐truxillic acids. The isolated yield of **2** is approximately 94%, which exceeds the theoretical maximum conversion rate of 86.5% for pure solid‐state photodimerization [30]. Therefore, phase rebuilding must have occurred and water may facilitate the crystalline‐state rearrangement of unreacted **14** [31, 32]. Consequently, suspension dimerization surpasses neat solid‐state dimerization due to continuously refreshed exposure and facilitated phase rebuilding.

**FIGURE 3:**
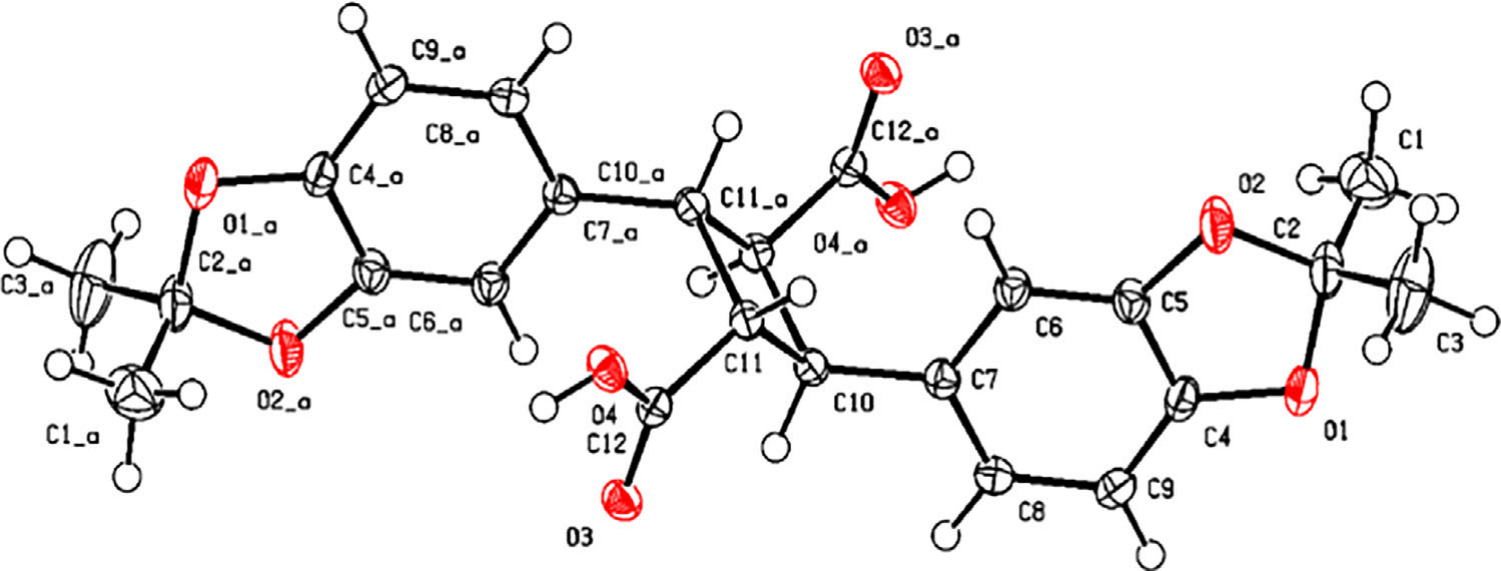
The structure of truxillic acid **2** by x‐ray diffraction.

### 2.4 Convergent Synthesis of SBA‐C and Its Methyl Ester

With both building blocks in hand, diacid **2** was condensed with the *D*‐DSS block **1** using Steglich esterification, with *N*,*N*′‐dicyclohexylcarbodiimide(DCC) as an activator and 4‐dimethylaminopyridine(DMAP) as a catalyst, affording the fully protected SBA‐C methyl ester (**15**) in 85% yield (Scheme 5). This result indicates that the two remotely located carboxyl groups are sufficiently reactive and do not interfere with each other. Subsequent alkaline hydrolysis of **15** with LiOH in THF/H_2_O selectively cleaved the methyl esters to give an acetonide‐protected SBA‐C (**16**) in 93% yield, confirming that the secondary alcoholic ester is more stable than the primary methyl ester.

**SCHEME 5:**
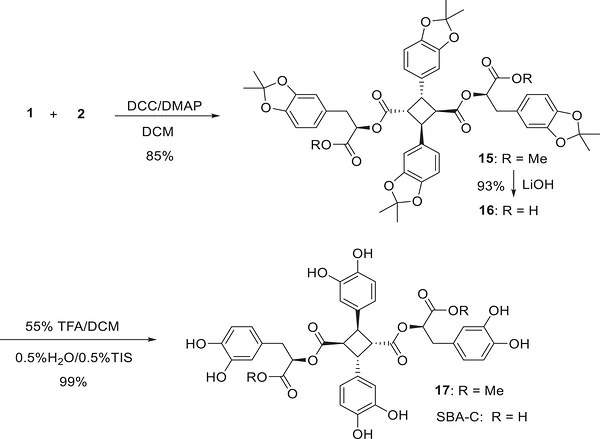
Synthetic route to SBA‐C and its methyl ester.

To establish selective deprotection conditions enabling rapid and complete removal of acetonide groups without affecting other labile bonds in **15** and **16**, a range of reaction systems was screened by RP‐HPLC. Catalysts including TFA, HCl, and AcOH were evaluated in solvents such as dichloromethane (DCM), THF, dioxane, and H_2_O. A 50–70% solution of TFA in DCM containing a trace amount of water was found to provide excellent selectivity.

Under optimized conditions on a 60 mg scale, the acetonide groups of **16** and **15** were cleanly removed within 2 h using 55 vol% TFA in DCM at 0 °C, in the presence of 0.5 vol% H_2_O and 0.5 vol% triisobutylsilane (TIS) as a cation scavenger. Following lyophilization, SBA‐C was characterized by HRMS and NMR spectroscopy, and the data were consistent with those reported for the natural product. The corresponding products—SBA‐C and its methyl ester (**17**)—were obtained in nearly quantitative yield with high purity (ca. 99%), as determined by RP‐HPLC analysis at 280 nm. The results demonstrate that the acetonide protection of catechol is more susceptible to acidic hydrolysis than secondary alcoholic esters and methyl esters. Selective deprotection of the acetonide can be achieved under very mild conditions via kinetic control, potentially minimizing or avoiding side reactions that would otherwise lead to significantly lower conversion rates [10].

## 3 Conclusion

The first synthesis of the natural product SBA‐C has been accomplished in seven steps from caffeic acid in 35% overall yield, including a three‐step synthesis of the acetonide‐protected *D*‐DSS building block in 60%. Direct acetonide protection of *D*‐DSS was impeded by an undesired oxa‐Pictet–Spengler reaction, necessitating pre‐protection of the α‐hydroxyl group. Consequently, the required *D*‐DSS block was obtained via methanolysis of bis‐acetonide‐protected methyl rosmarinate. Instead of just standing by, acetonide protecting groups direct photodimerization toward *α*‐truxillic acids by actively modulating the molecular packing arrangement. Acetonide‐protection of catechol can be selectively c in nearly quantitative yield under mild conditions without causing significant side reactions, thus affording SBA‐C in high yield and purity. These results strongly support the application of the acetonide protection strategy for the synthesis of *D*‐DSS‐containing phenolic acids, such as salvianolic acids. Further work on their synthesis is underway.

## 4 Experimental Section

### 4.1 General Methods

All reagents were purchased from commercial suppliers (Sigma–Aldrich, Alfa Aesar, Xi'an Changhong Biotechnology Co., Ltd.) and used without further purification. ^1^H and ^13^C NMR spectra were recorded on a Bruker Avance 400 MHz spectrometer. High‐Resolution Mass Spectra (HRMS) were acquired using an LCMS‐IT‐TOF mass spectrometer (Shimadzu). Analytical TLC was performed on pre‐coated silica gel plates. Flash column chromatography was carried out using silica gel (300‐400 mesh). HPLC analyses were performed on a Waters 2695‐2998 system equipped with a Symmetry C18 column (4.6 × 250 mm, 5 µm) using a gradient of solvent A (water/acetonitrile/TFA, 89.9:10:0.1) and solvent B (acetonitrile/water/TFA, 89.9:10:0.1) at a flow rate of 1.0 mL/min with UV detection at 280/215 nm.

### 4.2 Synthetic Procedures

#### 4.2.1 Synthesis of Methyl (*R*)‐2,2,5,5‐tetramethyl‐7,8‐dihydro‐5*H*‐[1,3]dioxolo[4,5‐*g*]isochromene‐7‐carboxylate (**5**)

Salvianolic acid B (60% purity) was converted into Salvianolic acid A and *D*‐DSS via high‐pressure hydrolysis. The resulting mixture was then treated with SOCl_2_/MeOH to produce a crude mixture containing *D*‑DSS methyl ester. This mixture underwent a ring‑closing reaction in the DMP/TsOH/PhH/acetone system, yielding colorless crystals. The structure of this compound was characterized as compound **5**. HPLC retention time (RT) 18.3 min. ^1^H NMR (400 MHz, CDCl_3_) δ 6.45 (s, 1H), 6.43 (s, 1H), 4.42 (dd, *J* = 11.4, 3.2 Hz, 1H), 3.79 (s, 3H), 2.94 (dd, *J* = 15.7, 11.4 Hz, 1H), 2.81 (dd, *J* = 15.7, 3.2 Hz, 1H), 1.64 (s, 6H), 1.55 (s, 3H), 1.49 (s, 3H). ^13^C NMR (101 MHz, CDCl_3_) δ 172.04, 146.47, 146.04, 134.71, 123.80, 117.93, 108.03, 105.10, 77.48, 77.16, 76.84, 76.74, 68.84, 52.33, 32.34, 31.39, 28.43, 25.94. ^13^C NMR (101 MHz, CDCl_3_) DEPT δ CH 108.03, 105.10, 68.83; CH_2_ 32.34; CH_3_ 52.33, 31.39, 28.42, 25.94. HRMS Calcd. for [M+Na]^+^, 315.1203, found 315.1201. The HPLC chromatogram, HRMS spectrum, and NMR spectra for compound **5** are provided in Figures S1, S16, and S30–S32, respectively.

#### 4.2.2 Synthesis of (*R*)‐3‐(3,4‐dihydroxyphenyl)‐1‐methoxy‐1‐oxopropan‐2‐yl (*E*)‐3‐(3,4‐dihydroxyphenyl)acrylate (**6**)

Rosmarinic acid (5.0 g, 13.8 mmol) was suspended in anhydrous methanol (25 mL) and DMP (100 mL). Me_3_SiCl (17.25 mL) was added dropwise at 0°C under an argon atmosphere. The reaction mixture was stirred at room temperature for 3 h (monitored by TLC). The solvent was removed under reduced pressure. The residue was dissolved in ethyl acetate (150 mL), washed with water (3 × 50 mL), dried over anhydrous MgSO_4_, filtered, and concentrated to afford compound **6** as a yellow solid (4.65 g, 90%). HPLC RT 11.4 min; HRMS Calcd. for C_19_H_18_O_8_ [M+Na]^+^ 397.0894, found 397.0898. The HPLC chromatogram and HRMS spectrum for compound **6** are provided in Figures S2 and S17, respectively.

#### 4.2.3 Synthesis of (*R*)‐3‐(2,2‐dimethylbenzo[*d*][1,3]dioxol‐5‐yl)‐1‐methoxy‐1‐oxopropan‐2‐yl (*E*)‐3‐(3,4‐dihydroxyphenyl)acrylate (**7**)

Compound **6** (1.46 g, 3.6 mmol) was dissolved in acetone (10 mL) and benzene (150 mL) in a 250 mL three‐necked flask equipped with a Soxhlet apparatus containing anhydrous CaCl_2_. The solution was heated to reflux under argon. DMP (0.88 mL, 7.2 mmol) and TsOH (15 mg, 0.09 mmol) were added. The reaction was refluxed for 1‐1.5 h. After TLC analysis indicated complete conversion, the mixture was cooled, concentrated, and purified by flash chromatography (ethyl acetate/hexane) to give compound **7** as a yellow oil (1.19 g, 80%). HPLC RT 17.1 min. ^1^H NMR (400 MHz, CD_3_OD) δ 7.56 (d, *J* = 15.8 Hz, 1H), 7.04 (d, *J* = 2.1 Hz, 1H), 6.94 (dd, *J* = 8.1, 2.1 Hz, 1H), 6.77 (d, *J* = 8.1 Hz, 1H), 6.70 – 6.63 (m, 3H), 6.25 (d, *J* = 15.8 Hz, 1H), 5.21 (dd, *J* = 7.6, 5.1 Hz, 1H), 3.71 (s, 3H), 3.11 (dd, *J* = 14.2, 5.1 Hz, 1H), 3.06 (dd, *J* = 14.2, 7.6 Hz, 1H), 1.62 (s, 3H), 1.61 (s, 3H). ^13^C NMR (101 MHz, CD_3_OD) δ 171.98, 168.23, 149.87, 148.82, 148.01, 147.83, 146.80, 130.31, 127.46, 123.24, 123.00, 118.97, 116.50, 115.24, 114.01, 110.50, 108.89, 74.50, 52.71, 38.09, 25.88. HRMS Calcd. for [M‐H]^−^ 413.1242, found 413.1257. The HPLC chromatogram, HRMS spectrum, and NMR spectra for compound **7** are provided in Figures S3, S18, and S33–S35, respectively.

#### 4.2.4 Synthesis of (*R*)‐3‐(2,2‐dimethylbenzo[*d*][1,3]dioxol‐5‐yl)‐1‐methoxy‐1‐oxopropan‐2‐yl (*E*)‐3‐(2,2‐dimethylbenzo[*d*][1,3]dioxol‐5‐yl)acrylate (**8**)

Compound **6** (1.46 g, 3.6 mmol) was dissolved in acetone (10 mL) and benzene (150 mL) in a 250 mL three‐necked flask equipped with a Soxhlet apparatus containing anhydrous CaCl_2_. The solution was heated to reflux under argon. DMP (3.5 mL, 28.8 mmol) and TsOH (0.12 g, 0.72 mmol) were added. The reaction was refluxed for 2‐5 h with azeotropic removal of byproducts. After completion (TLC), the mixture was cooled, concentrated, and purified by flash chromatography (ethyl acetate/hexane) to give compound **8** as a yellow oil (1.37 g, 83%). HPLC RT 22.2 min. ^1^H NMR (400 MHz, CDCl_3_) δ 7.60 (d, *J* = 15.9 Hz, 1H), 7.00– 6.93 (m, 2H), 6.71 (d, *J* = 7.9 Hz, 1H), 6.66 ‐6.64(m, 3H), 6.27 (d, *J* = 15.9 Hz, 1H), 5.27 (dd, *J* = 7.7, 4.8 Hz, 1H), 3.74 (s, 3H), 3.12(dd, *J* = 14.1, 4.8 Hz, 1H), 3.07(dd, *J* = 14.1, 7.7 Hz, 1H), 1.68 (s, 6H), 1.654 (s,3H), 1.648 (s,3H). ^13^C NMR (101 MHz, CDCl_3_) δ 170.48, 166.60, 149.93, 148.28, 147.59, 146.57, 146.26, 129.04, 128.16, 124.54, 121.93, 119.09, 117.98, 114.39, 109.64, 108.49, 108.15, 106.54, 73.26, 52.43, 37.38, 26.00, 25.96. HRMS Calcd. for C_25_H_26_O_8_ [M+Na]^+^, 477.1520, found 477.1535. The HPLC chromatogram, HRMS spectrum, and NMR spectra for compound **8** are provided in Figures S4, S19, S36, and S37, respectively.

#### 4.2.5 Synthesis of (*R,E*)‐3‐(2,2‐dimethylbenzo[*d*][1,3]dioxol‐5‐yl)‐2‐((3‐(2,2‐dimethylbenzo[*d*][1,3]dioxol‐5‐yl)acryloyl)oxy)propanoic acid (**9**)

Compound **8** (0.26 g, 0.57 mmol) was dissolved in THF (12 mL) and water (6 mL) in a 50 mL single‑necked flask. The mixture was cooled in an ice bath, and a solution of LiOH (0.027 g, 1.14 mmol) was added dropwise. The reaction mixture was stirred for 2 h and monitored by TLC. After completion, the mixture was quenched with saturated ammonium chloride solution and extracted with ethyl acetate. The organic phase was washed with water, dried over anhydrous MgSO_4_, filtered, and concentrated under reduced pressure. The residue was purified by column chromatography to afford the target compound as a yellow solid (0.24 g, 98%). HPLC RT 19.7 min. ^1^H NMR (400 MHz, CDCl_3_) δ 7.61 (d, *J* = 15.9 Hz, 1H), 6.96 (d, *J* = 8.7 Hz, 2H), 6.67 (dt, *J* = 16.7, 9.3 Hz, 4H), 6.25 (t, *J* = 14.9 Hz, 2H), 5.29 (d, *J* = 4.2 Hz, 1H), 3.14 (m, *J* = 22.8, 14.4, 6.0 Hz, 2H), 1.73–1.56 (m, 12H). ^13^C NMR (101 MHz, CDCl_3_) δ 174.54, 166.77, 149.95, 148.26, 147.57, 146.57, 146.52, 129.02, 128.14, 124.63, 122.01, 119.10, 118.00, 114.22, 109.69, 108.48, 108.20, 106.61, 77.16, 73.12, 37.15, 25.95. HRMS Calcd. for [M‐H]^−^ 439.1398, found 439.1416. The HPLC chromatogram, HRMS spectrum, and NMR spectra for compound **9** are provided in Figures S5, S20, and S38–S40, respectively.

#### 4.2.6 Synthesis of a mixture of Methyl (*Z*)‐3‐(2,2‐dimethylbenzo[*d*][1,3]dioxol‐5‐yl)acrylate and methyl (*E*)‐3‐(2,2‐dimethylbenzo[*d*][1,3]dioxol‐5‐yl)acrylate (**11**)

Compound **8** (0.454 g, 1.0 mmol) was dissolved in anhydrous methanol (10 mL) and cooled to 0°C. Sodium methoxide (NaOMe, 0.54 g, 10.0 mmol) was added. The reaction mixture was stirred at 0°C for 1 h, then warmed to room temperature and stirred overnight (monitored by TLC). The reaction was quenched with saturated NH_4_Cl solution. The mixture was extracted with ethyl acetate (3 × 30 mL). The combined organic layers were washed with water, dried over MgSO_4_, concentrated, and purified by flash chromatography to afford compound **11** as a yellow oily residue. HPLC RT 18.7 min. ^1^H NMR (400 MHz, CDCl_3_) δ major isomer: 7.58 (d, *J* = 15.9 Hz, 1H), 6.98–6.70 (m, 3H), 6.23 (d, *J* = 15.9 Hz, 1H), 3.77 (s, 3H), 1.68 (s, 6H); minor isomer: 7.38 (s, 1H), 7.07–6.96 (m, 3H), 5.78 (d, *J* = 12.8 Hz, 1H), 3.72 (s, 3H), 1.67 (s, 6H). ^1^ ^3^C NMR (101 MHz, CDCl_3_) δ major isomer: 168.28, 150.14, 148.70, 145.46, 128.76, 124.64, 119.45, 115.67, 108.92, 106.81, 52.12, 26.43; minor isomer: 167.34, 149.13, 147.86, 144.19, 126.16, 119.04, 116.99, 110.52, 108.24, 51.87. HRMS Calcd. for C_13_H_14_O_4_ [M+H]^+^, 235.0965, found 235.0966. The HPLC chromatogram, HRMS spectrum, and NMR spectra for compound **11** are provided in Figures S6, S21, S41 and S42, respectively.

#### 4.2.7 Synthesis of Methyl (*R*)‐3‐(2,2‐dimethylbenzo[*d*][1,3]dioxol‐5‐yl)‐2‐hydroxypropanoate (**1**)

Compound **8** (0.454 g, 1.0 mmol) was dissolved in anhydrous methanol (10 mL) and cooled to 0°C. Sodium methoxide (NaOMe, 0.54 g, 1.0 mmol) was added. The reaction was stirred at 0°C for 1 h (monitored by TLC) and then quenched with saturated NH_4_Cl solution. The mixture was extracted with ethyl acetate (3 × 30 mL). The combined organic layers were washed with water, dried over MgSO_4_, concentrated, and purified by flash chromatography (ethyl acetate/petroleum ether = 1:5) to afford compound **1** as a yellow solid (0.20 g, 80%). HPLC RT 14.2 min. ^1^H NMR (400 MHz, CD_3_OD) δ 6.63 (d, *J* = 1.3 Hz, 1H), 6.61 (m, 2H), 4.30 (dd, *J* = 7.6, 4.9 Hz, 1H), 3.69 (s, 3H), 2.95 (dd, *J* = 14.0, 4.9 Hz, 1H), 2.81 (dd, *J* = 14.0, 7.6 Hz, 1H), 1.61 (s, 6H). ^13^C NMR (101 MHz, CD_3_OD) δ 175.69, 148.67, 147.51, 131.48, 122.93, 118.71, 110.57, 108.73, 73.17, 52.34, 41.30, 25.90. ^13^C NMR (101 MHz, CD_3_OD) DEPT δ CH 122.92, 110.56, 108.73, 73.16, 52.33; CH_2_ 41.41; CH_3_ 25.90. HRMS Calcd. for C_13_H_16_O_5_ [M+Na]^+^, 275.0890, found 275.0885. The HPLC chromatogram, HRMS spectrum, and NMR spectra for compound **1** are provided in Figures S7, S22, and S43–S45, respectively.

#### 4.2.8 Synthesis of Methyl (E)‐3‐(3,4‐dihydroxyphenyl)acrylate (**12**)

Caffeic acid (9.07 g, 50 mmol) was suspended in anhydrous methanol (150 mL) in a 500 mL two‐necked flask under argon. Thionyl chloride (SOCl_2_, 15.5 mL, 200 mmol) was added dropwise at 0°C. The mixture was stirred at room temperature for 10‐24 h (monitored by HPLC/TLC). The solvent was removed under reduced pressure to give compound **12** as a gray solid (9.13 g, 94%). HPLC RT 10.8 min. The HPLC chromatogram for compound **12** is provided in Figure S8.

#### 4.2.9 Synthesis of Methyl (*E*)‐3‐(2,2‐dimethylbenzo[*d*][1,3]dioxol‐5‐yl)acrylate (**13**)

Compound **12** (5.82 g, 30 mmol) was dissolved in dry benzene (200 mL) and dry acetone (90 mL). The solution was heated to reflux under argon. DMP (15 mL, 120 mmol) and TsOH (0.258 g, 1.5 mmol) were added. The reaction was refluxed for 2‐4 h (monitored by TLC/HPLC). After cooling, the mixture was concentrated and purified by flash chromatography (DCM/petroleum ether) to give compound **13** as a sticky residue (6.18 g, 88%). HPLC RT 18.6 min. ^1^H NMR (400 MHz, CDCl_3_) δ 7.58 (d, *J* = 15.9 Hz, 1H), 6.94 (d, *J* = 7.5 Hz, 2H), 6.70 (d, *J* = 7.7 Hz, 1H), 6.23 (d, *J* = 15.9 Hz, 1H), 1.67 (s, 6H). ^13^C NMR (101 MHz, CDCl_3_) δ 168.24, 150.12, 148.69, 145.42, 128.75, 124.62, 119.42, 115.66, 108.89, 106.80, 77.60, 52.08, 26.40. HRMS Calcd. for C_13_H_14_O_4_ [M+H]^+^, 235.0965, found 235.0966. The HPLC chromatogram, HRMS spectrum, and NMR spectra for compound **13** are provided in Figures S9, S23, S46, and S47, respectively.

#### 4.2.10 Synthesis of (*E*)‐3‐(2,2‐dimethylbenzo[*d*][1,3]dioxol‐5‐yl)acrylic acid (**14**)

Compound **13** (2.34 g, 10 mmol) was dissolved in THF (30 mL). A solution of LiOH (0.48 g, 20 mmol) in water (15 mL) was added. The reaction was stirred at room temperature for 3 h (monitored by TLC). The THF was removed under reduced pressure. The aqueous phase was washed with ethyl acetate (2 × 50 mL), acidified to pH ∼3 with 2 M HCl, and extracted with ethyl acetate (3 × 20 mL). The combined organic extracts were dried over MgSO_4_ and concentrated to give compound **14** as a pale‐yellow solid (2.10 g, 95%). HPLC RT 14.6 min. ^1^H NMR (400 MHz, CDCl_3_) δ 7.68 (d, *J* = 15.8 Hz, 1H), 6.98 (d, *J* = 9.4 Hz, 2H), 6.73 (d, *J* = 7.8 Hz, 1H), 6.24 (d, *J* = 15.8 Hz, 1H), 1.69 (s, 6H). ^13^C NMR (101 MHz, CDCl_3_) δ 172.99, 150.03, 148.23, 147.14, 128.33, 124.76, 119.63, 115.24, 108.54, 106.60, 77.16, 25.83. HRMS Calcd. for C_12_H_12_O_4_ [M+H]^+^, 221.0808, found 221.0809. The HPLC chromatogram, HRMS spectrum, NMR spectra, and single‐crystal XRD data for compound **14** are provided in Figures S10, S24, S48, S49, and S67, respectively.

#### 4.2.11 Synthesis of (1*α*, 2*α*, 3*β*, 4*β*)‐2,4‐bis(2,2‐dimethylbenzo[*d*][1,3]dioxol‐5‐yl)cyclobutane‐1,3‐dicarboxylic acid (*α*‐Truxillic Acid Derivative, **2**)

The photodimerization setups for small‐scale and large‐scale reactions are shown in Figures S65 and S66, respectively.

Compound **14** (0.11 g, 0.5 mmol) was suspended in a mixture of absolute ethanol (0.25 mL) and pure water (4.75 mL) in a 10 mL round‐bottom flask. The suspension was stirred at room temperature for 25 h under irradiation from a black light lamp (320–400 nm) placed at a distance of 2 cm, and the reaction was monitored by HPLC/TLC. The precipitate was collected by filtration. The crude solid was recrystallized from absolute ethanol to afford compound **2** as white crystals (0.103 g, 94%). Single crystals suitable for x‐ray diffraction were obtained by slow evaporation from ethanol. HPLC RT 15.4 min. ^1^H NMR (400 MHz, DMSO‐*d*_6_) δ 6.83 (s, 2H), 6.79 – 6.66 (m, 4H), 4.15 (dd, *J* = 10.3, 7.4 Hz, 2H), 3.67 (dd, *J* = 10.3, 7.3 Hz, 2H), 1.62 (s, 12H). ^13^C NMR (101 MHz, DMSO‐*d*_6_) δ 173.08(2C), 146.77(2C), 145.66(2C), 132.77(2C), 120.43(2C), 117.78(2C), 107.98(2C), 107.71(2C), 46.69(2C), 40.81(2C)25.62(4C). HRMS Calcd. for C_24_H_24_O_8_ [M‐H]^−^, 439.1398, found 439.1394. The HPLC chromatogram, HRMS spectrum, NMR spectra, and single‐crystal XRD data for compound **2** are provided in Figures S11, S25, S50–S52, and S68, respectively.

#### 4.2.12 Synthesis of bis((*R*)‐3‐(2,2‐dimethylbenzo[*d*][1,3]dioxol‐5‐yl)‐1‐methoxy‐1‐oxopropan‐2‐yl)‐(1*α*, 2*α*, 3*β*, 4*β*)‐2,4‐bis(2,2‐dimethylbenzo[*d*][1,3]dioxol‐5‐yl)cyclobutane‐1,3‐dicarboxylate (**15**)

Compound **1** (0.75 g, 3.0 mmol) and compound **2** (0.66 g, 1.5 mmol) were dissolved in dry DCM (100 mL). DMAP (0.75 g, 6.0 mmol) and DCC (0.74 g, 3.6 mmol) were added. The reaction was stirred at room temperature for 4 h (monitored by HPLC/TLC). The precipitate dicyclohexylurea (DCU) was removed by filtration. The filtrate was washed with water, dried over MgSO_4_, concentrated, and purified by flash chromatography (DCM) to give compound **15** as a white solid (1.16 g, 85%). HPLC RT 25.1 min. ^1^H NMR (400 MHz, CDCl_3_) δ 6.64 – 6.41 (m, 12H), 4.91 (dd, *J* = 7.8, 5.3 Hz, 1H), 4.76 (t, *J* = 6.1 Hz, 1H), 4.31 – 4.22 (m, 2H), 3.90 – 3.83 (m, 2H), 3.59 (d, *J* = 14.4 Hz, 6H), 2.88 (dd, *J* = 22.0, 14.1, 6.6 Hz, 2H), 2.76 – 2.61 (m, 2H), 1.63 (t, *J* = 8.4 Hz, 24H). ^13^C NMR (101 MHz, CDCl_3_) δ 171.52, 170.83, 169.86, 169.60, 147.69, 147.64, 147.62, 147.50, 146.66, 146.59, 146.57, 146.56, 131.60, 131.48, 128.67, 128.61, 128.38, 128.33, 121.96, 121.92, 120.23, 119.96, 118.11, 117.99, 117.96, 109.65, 109.54, 108.25, 108.20, 108.10, 107.99, 107.74, 73.48, 73.44, 52.23(2C), 47.07, 46.98, 41.77, 41.37, 37.43, 37.06, 25.97(4C), 25.93(4C). HRMS Calcd. for C_50_H_52_O_16_ [M+Na]^+^ 931.3148, found 931.3190. The HPLC chromatogram, HRMS spectrum, and NMR spectra for compound **15** are provided in Figures S12, S26, and S53–S55, respectively.

#### 4.2.13 Synthesis of (2*R*,2'*R*)‐2,2'‐(((1*α*, 2*α*, 3*β*, 4*β*)‐2,4‐bis(2,2‐dimethylbenzo[*d*][1,3]dioxol‐5‐yl)cyclobutane‐1,3‐dicarbonyl)bis(oxy))bis(3‐(2,2‐dimethylbenzo[*d*][1,3]dioxol‐5‐yl)propanoic acid) (**16**)

Compound **15** (0.54 g, 0.59 mmol) was dissolved in THF (24 mL) and water (12 mL). The solution was cooled in an ice bath. A solution of LiOH (0.086 g, 3.6 mmol) in water (12 mL) was added dropwise over 30 min. The reaction was stirred for 3 h 40 min (monitored by HPLC/TLC). The reaction was quenched with 0.5 M citric acid solution and the pH of the mixture was adjusted to approximately 4. The mixture was extracted with ethyl acetate (3 × 50 mL). The combined organic layers were washed with water, dried over MgSO_4_, concentrated, and purified by flash chromatography (ethyl acetate/petroleum ether) to give compound **16** as a white solid (0.47 g, 93%). HPLC RT 21.3 min. ^1^H NMR (400 MHz, CDCl_3_) δ 6.62 – 6.45 (m, 12H), 4.96 – 4.72 (m, 2H), 4.33 – 4.18 (m, 2H), 3.85 (dd, *J* = 17.1, 9.9 Hz, 2H), 2.87 (dd, *J* = 14.1, 5.1 Hz, 1H), 2.81 (dd, *J* = 14.1, 7.7 Hz, 1H), 2.72 (dd, *J* = 14.1, 6.2 Hz, 1H), 2.60 (dd, *J* = 14.1, 5.7 Hz, 1H). ^13^C NMR (101 MHz, CDCl_3_) δ 174.34, 173.91, 171.53, 170.81, 147.68, 147.63(2C), 147.49, 146.65(2C), 146.64, 146.60, 131.49, 131.20, 128.50, 128.27, 122.03(2C), 120.19, 119.96, 118.16, 118.06(2C), 118.02, 109.73, 109.58, 108.29, 108.18(4C), 107.66, 73.08, 73.02, 47.02, 46.91, 41.72, 41.44, 37.21, 36.83, 26.01(2C), 25.94(4C), 25.86(2C). HRMS Calcd. for C_48_H_48_O_16_[M‐H]^−^, 879.2870, found 879.2872. The HPLC chromatogram, HRMS spectrum, and NMR spectra for compound **16** are provided in Figures S13, S27, and S56–S58, respectively.

#### 4.2.14 Synthesis of bis((*R*)‐3‐(3,4‐dihydroxyphenyl)‐1‐methoxy‐1‐oxopropan‐2‐yl)‐ (1*α*, 2*α*, 3*β*, 4*β*)‐2,4‐bis(3,4‐dihydroxyphenyl)cyclobutane‐1,3‐dicarboxylate (**17**)

Compound **15** (60 mg, 0.066 mmol) and a magnetic stir bar were placed in a 10 mL single‑neck flask. Deprotection solution (TFA/DCM/H_2_O/TIS = 55:44:0.5:0.5, 6 mL) was added. The reaction was stirred under ice‑bath conditions (monitored by HPLC). After 2 h, the reaction was complete. The solvent was removed by rotary evaporation. Water was added, and the mixture was placed in a −80°C freezer. After being completely frozen, it was lyophilized for 48 h to give a white solid (48 mg, 99%). HPLC RT 11.4 min. ^1^H NMR (400 MHz, CD_3_OD) δ 6.77 – 6.37 (m, 12H), 4.93 (s, 8H), 4.77 (d, *J* = 6.2 Hz, 1H), 4.56 (t, *J* = 6.2 Hz, 1H), 4.25 – 4.15 (m, 2H), 3.88 (dd, *J* = 10.6, 7.2 Hz, 1H), 3.77 (dd, *J* = 10.5, 7.0 Hz, 1H), 3.57 (t, *J* = 14.4 Hz, 6H), 2.86 – 2.75 (m, 2H), 2.62 (dd, *J* = 38.7, 14.0, 6.2 Hz, 2H). ^13^C NMR (101 MHz, CD_3_OD) δ 173.21, 172.62, 171.95, 171.47, 146.18, 146.16, 146.03, 145.99, 145.50, 145.35, 145.29, 145.28, 131.47, 131.37, 128.34, 128.08, 122.01, 120.37, 119.66, 117.54, 117.40, 116.40, 116.36, 116.31, 116.27, 115.96, 115.54, 74.94, 52.65, 52.56, 49.00, 48.32, 48.00, 42.66, 42.08, 37.88, 37.58. HRMS Calcd. for [M‐H]^−^, 747.1931, found 747.1937. The HPLC chromatogram, HRMS spectrum, and NMR spectra for compound **17** are provided in Figures S14, S28, and S59–S61, respectively.

#### 4.2.15 Synthesis of Shimobashiric Acid C

Compound **16** (60 mg, 0.066 mmol) was placed in a 10 mL round‐bottom flask. A pre‐cooled (0°C) deprotection mixture of TFA/DCM/H_2_O/TIS (55:44:0.5:0.5, 6 mL) was added. The reaction was stirred at 0°C for 4 h (monitored by HPLC). The solvent was removed under reduced pressure. Water was added, and the solution was frozen at −80°C and lyophilized for 24 h to afford Shimobashiric acid C as a white solid (48.5 mg, 99%). Purity: >99% (HPLC at 280 nm). HPLC RT 10.6 min. ^1^H NMR (400 MHz, CD_3_OD) δ 6.78 (d, *J* = 2.0 Hz, 1H), 6.70 (d, *J* = 8.0 Hz, 1H), 6.68 (d, *J* = 8.0 Hz, 1H), 6.67 (d, *J* = 8.0 Hz, 1H), 6.66 (d, *J* = 2.0 Hz, 1H), 6.66 (d, *J* = 2.0 Hz, 1H), 6.64 (d, *J* = 8.0 Hz, 1H), 6.63 (d, *J* = 2.0 Hz, 1H), 6.53 (dd, *J* = 8.0, 2.0 Hz, 1H), 6.48 (dd, *J* = 8.0, 2.0 Hz, 1H), 6.45 (dd, *J* = 8.0, 2.0 Hz, 1H), 6.35 (dd, *J* = 8.0, 2.0 Hz, 1H), 4.72 (dd, *J* = 7.3, 5.2 Hz, 1H), 4.57 (t, *J* = 5.9 Hz, 1H), 4.25 – 4.14 (m, 2H), 3.91 (dd, *J* = 10.5, 7.5 Hz, 1H), 3.75 (dd, *J* = 10.2, 6.8 Hz, 1H), 2.87 (dd, *J* = 14.1, 5.1 Hz, 1H), 2.81 (dd, *J* = 14.1, 7.7 Hz, 1H), 2.72 (dd, *J* = 14.1, 6.2 Hz, 1H), 2.60 (dd, *J* = 14.1, 5.7 Hz, 1H). ^13^C NMR (101 MHz, CD_3_OD) δ 173.37, 173.29, 172.83, 172.70, 146.15, 146.09, 145.93, 145.87, 145.40, 145.26, 145.16, 145.11, 131.62, 131.50, 128.83, 128.53, 122.18, 122.15, 120.51, 119.34, 117.77, 117.49, 116.46, 116.39(2C), 116.24, 115.98, 115.49, 74.91, 74.81, 48.57, 47.88, 42.74, 41.99, 37.80, 37.48. HRMS Calcd. for C_36_H_32_O_16_ [M‐H]^−^, 719.1618, found 719.1618. The HPLC chromatogram, HRMS spectrum, and NMR spectra of synthetic SBA‐C are provided in Figures S15, S29, and S62–S64, respectively. The ^1^H and ^13^C NMR data of synthetic SBA‐C agree well with those of the natural product, as detailed in Tables S1 and S2.

## Author Contributions

Ruwen Li wrote this manuscript and carried out most of the synthesis; Zhe Zhang large‐scale photodimerization and crystal growth of **14**; Pengshu Xu, Synthesis of **8**; Lin Zhou and Lei Chen, help with the synthesis of **1** and **9**; Zhongqiang Liu designed the project and edited the manuscript.

## Conflicts of Interest

A patent application (CN 202610772580.9) related to this work has been filed, with Zhongqiang Liu, Ruwen Li, Pengshu Xu, Lei Chen, and Lin Zhou listed as co‐inventors.

## Supporting information



Deposition Number(s) 2560057 (for **14**), 2546543 (for **2**) contain(s) the supplementary crystallographic data for this paper. These data are provided free of charge by the joint Cambridge Crystallographic Data Centre and Fachinformationszentrum Karlsruhe Access Structures service.
**Supporting File 1**: cbdv71738‐sup‐0001‐SuppMat.docx

## Data Availability

The data that support the findings of this study are available in the Supporting Information of this article.
