# Supplementary material for: First Synthesis of Shimobashiric Acid C With Acetonide as the Catechol Protecting Group
Source: Chem Biodivers. 2026 Sep 30;23(10):e71738. doi: 10.1002/cbdv.71738 (PMC13626253; doi:10.1002/cbdv.71738)
Supplement: Supplementary file 1 [file CBDV-23-e71738-s001.docx]

**Supporting Information**

**for**

**First Synthesis of Shimobashiric Acid C with Acetonide as the Catechol Protecting Group**

Ruwen Li,^1^ Zhe Zhang,^1^ Pengshu Xu,^1^ Lin Zhou,^1^ Lei Chen,^1^ Zhongqiang Liu*^1^

^1^School of Pharmaceutical Sciences, Hainan University, 58 Renmin Avenue, Meilan District, Haikou 570228, Hainan Province, P. R. China.

* Corresponding authors.

Dr. Zhongqiang Liu, E-mail:zqliu@hainanu.edu.cn

**Contents**

[Figure S1. HPLC spectrum of compound **5**	1](#_Toc232367740)

[Figure S2. HPLC spectrum of compound **6**	1](#_Toc232367741)

[Figure S3. HPLC spectrum of compound **7**	2](#_Toc232367742)

[Figure S4. HPLC spectrum of compound **8**	2](#_Toc232367743)

[Figure S5. HPLC spectrum of compound **9**	3](#_Toc232367744)

[Figure S6. HPLC spectrum of compound **11**	3](#_Toc232367745)

[Figure S7. HPLC spectrum of compound **1**	4](#_Toc232367746)

[Figure S8. HPLC spectrum of compound **12**	4](#_Toc232367747)

[Figure S9. HPLC spectrum of compound **13**	5](#_Toc232367748)

[Figure S10. HPLC spectrum of compound **14**	5](#_Toc232367749)

[Figure S11. HPLC spectrum of compound **2**	6](#_Toc232367750)

[Figure S12. HPLC spectrum of compound **15**	6](#_Toc232367751)

[Figure S13. HPLC spectrum of compound **16**	7](#_Toc232367752)

[Figure S14. HPLC spectrum of compound **17**	7](#_Toc232367753)

[Figure S15. HPLC spectrum of SBA-C	8](#_Toc232367754)

[Figure S16. HR-MS spectrum of compound **5**	9](#_Toc232367755)

[Figure S17. HR-MS spectrum of compound **6**	9](#_Toc232367756)

[Figure S18. HR-MS spectrum of compound **7**	10](#_Toc232367757)

[Figure S19. HR-MS spectrum of compound **8**	10](#_Toc232367758)

[Figure S20. HR-MS spectrum of compound **9**	11](#_Toc232367759)

[Figure S21. HR-MS spectrum of compound **11**	11](#_Toc232367760)

[Figure S22. HR-MS spectrum of compound **1**	12](#_Toc232367761)

[Figure S23. HR-MS spectrum of compound **13**	12](#_Toc232367762)

[Figure S24. HR-MS spectrum of compound **14**	13](#_Toc232367763)

[Figure S25. HR-MS spectrum of compound **2**	13](#_Toc232367764)

[Figure S26. HR-MS spectrum of compound **15**	14](#_Toc232367765)

[Figure S27. HR-MS spectrum of compound **16**	14](#_Toc232367766)

[Figure S28. HR-MS spectrum of compound **17**	15](#_Toc232367767)

[Figure S29. HR-MS spectrum of SBA-C	15](#_Toc232367768)

[Figure S30. ^1^H NMR spectrum of compound **5** (400 MHz, CDCl_3_)	16](#_Toc232367769)

[Figure S31. ^13^C NMR spectrum of compound **5** (101 MHz, CDCl_3_)	16](#_Toc232367770)

[Figure S32. ^13^C DEPT spectrum of compound **5** (101 MHz, CDCl_3_)	17](#_Toc232367771)

[Figure S33. ^1^H NMR spectrum of compound **7** (400 MHz, CD_3_OD)	17](#_Toc232367772)

[Figure S34. ^13^C NMR spectrum of compound **7** (101 MHz, CD_3_OD)	18](#_Toc232367773)

[Figure S35. ^13^C DEPT spectrum of compound **7** (101 MHz, CD_3_OD)	18](#_Toc232367774)

[Figure S36. ^1^H NMR spectrum of compound **8** (400 MHz, CDCl_3_)	19](#_Toc232367775)

[Figure S37. ^13^C NMR spectrum of compound **8** (101 MHz, CDCl_3_)	19](#_Toc232367776)

[Figure S38. ^1^H NMR spectrum of compound **9** (400 MHz, CDCl_3_)	20](#_Toc232367777)

[Figure S39. ^13^C NMR spectrum of compound **9** (101 MHz, CDCl_3_)	20](#_Toc232367778)

[Figure S40. ^13^C DEPT spectrum of compound **9** (101 MHz, CDCl_3_)	21](#_Toc232367779)

[Figure S41. ^1^H NMR spectrum of compound **11** (400 MHz, CDCl_3_)	21](#_Toc232367780)

[Figure S42. ^13^C NMR spectrum of compound **11** (101 MHz, CDCl_3_)	22](#_Toc232367781)

[Figure S43. ^1^H NMR spectrum of compound **1** (400 MHz, CDCl_3_)	22](#_Toc232367782)

[Figure S44. ^13^C NMR spectrum of compound **1** (101 MHz, CDCl_3_)	23](#_Toc232367783)

[Figure S45. ^13^C DEPT spectrum of compound **1** (101 MHz, CDCl_3_)	23](#_Toc232367784)

[Figure S46. ^1^H NMR spectrum of compound **13** (400 MHz, CDCl_3_)	24](#_Toc232367785)

[Figure S47. ^13^C NMR spectrum of compound **13** (101 MHz, CDCl_3_)	24](#_Toc232367786)

[Figure S48. ^1^H NMR spectrum of compound **14** (400 MHz, CDCl_3_)	25](#_Toc232367787)

[Figure S49. ^13^C NMR spectrum of compound **14** (101 MHz, CDCl_3_)	25](#_Toc232367788)

[Figure S50. ^1^H NMR spectrum of compound **2** (400 MHz, DMSO-*d*_6_)	26](#_Toc232367789)

[Figure S51. ^13^C NMR spectrum of compound **2** (101 MHz, DMSO-*d*_6_)	26](#_Toc232367790)

[Figure S52. ^13^C DEPT spectrum of compound **2** (101 MHz, DMSO-*d_6_*)	27](#_Toc232367791)

[Figure S53. ^1^H NMR spectrum of compound **15** (400 MHz, CDCl_3_)	27](#_Toc232367792)

[Figure S54. ^13^C NMR spectrum of compound **15** (101 MHz, CDCl_3_)	28](#_Toc232367793)

[Figure S55. ^13^C DEPT spectrum of compound **15** (101 MHz, CDCl_3_)	28](#_Toc232367794)

[Figure S56. ^1^H NMR spectrum of compound **16** (400 MHz, CDCl_3_)	29](#_Toc232367795)

[Figure S57. ^13^C NMR spectrum of compound **16** (101 MHz, CDCl_3_)	30](#_Toc232367796)

[Figure S58. ^13^C DEPT spectrum of compound **16** (101 MHz, CDCl_3_)	30](#_Toc232367797)

[Figure S59. ^1^H NMR spectrum of compound **17** (400 MHz, CD_3_OD)	30](#_Toc232367798)

[Figure S60. ^13^C NMR spectrum of compound **17** (101 MHz, CD_3_OD)	31](#_Toc232367799)

[Figure S61. ^13^C DEPT spectrum of compound **17** (101 MHz, CD_3_OD)	31](#_Toc232367800)

[Figure S62. ^1^H NMR spectrum of SBA-C (400 MHz, CDCl_3_)	32](#_Toc232367801)

[Figure S63. ^13^C NMR spectrum of SBA-C (101 MHz, CDCl_3_)	32](#_Toc232367802)

[Figure S64. ^13^C DEPT spectrum of SBA-C (101 MHz, CDCl_3_)	33](#_Toc232367803)

[Table S1. Comparison of the ¹H NMR data of the synthetic and the natural SBA-C	34](#_Toc232367804)

[Table S2. Comparison of the ^13^C NMR data of the synthetic and the natural SBA-C	35](#_Toc232367805)

[Figure S65. Reconstruction of the small-scale experimental setup	36](#_Toc232367806)

[Figure S66. Snapshot and devices of the large-scale experimental setup	37](#_Toc232367807)

[Figure S67. Single crystal XRD spectrum of compound **14**	40](#_Toc232367808)

[Figure S68. Single crystal XRD spectrum of compound **2**	42](#_Toc232367809)

**Figure S1.** HPLC spectrum of compound **5**

**Figure S2.** HPLC spectrum of compound **6**

**Figure S3.** HPLC spectrum of compound **7**

**Figure S4.** HPLC spectrum of compound **8**

**Figure S5.** HPLC spectrum of compound **9**

**Figure S6.** HPLC spectrum of compound **11**

**Figure S7.** HPLC spectrum of compound **1**

**Figure S8.** HPLC spectrum of compound **12**

**Figure S9.** HPLC spectrum of compound **13**

**Figure S10.** HPLC spectrum of compound **14**

**Figure S11.** HPLC spectrum of compound **2**

**Figure S12.** HPLC spectrum of compound **15**

**Figure S13.** HPLC spectrum of compound **16**

**Figure S14.** HPLC spectrum of compound **17**

**Figure S15.** HPLC spectrum of SBA-C

**Figure S16.** HR-MS spectrum of compound **5**

**Figure S17.** HR-MS spectrum of compound **6**

**Figure S18.** HR-MS spectrum of compound **7**

**Figure S19.** HR-MS spectrum of compound **8**

**Figure S20.** HR-MS spectrum of compound **9**

**Figure S21.** HR-MS spectrum of compound **11**

**Figure S22.** HR-MS spectrum of compound **1**

**Figure S23.** HR-MS spectrum of compound **13**

**Figure S24.** HR-MS spectrum of compound **14**

**Figure S25.** HR-MS spectrum of compound **2**

**Figure S26.** HR-MS spectrum of compound **15**

**Figure S27.** HR-MS spectrum of compound **16**

**Figure S28.** HR-MS spectrum of compound **17**

**Figure S29.** HR-MS spectrum of SBA-C

**Figure S30.** ^1^H NMR spectrum of compound **5** (400 MHz, CDCl_3_)

**Figure S31.** ^13^C NMR spectrum of compound **5** (101 MHz, CDCl_3_)

**Figure S32.** ^13^C DEPT spectrum of compound **5** (101 MHz, CDCl_3_)

**Figure S33.** ^1^H NMR spectrum of compound **7** (400 MHz, CD_3_OD)

**Figure S34.** ^13^C NMR spectrum of compound **7** (101 MHz, CD_3_OD)

**Figure S35.** ^13^C DEPT spectrum of compound **7** (101 MHz, CD_3_OD)

**Figure S36.** ^1^H NMR spectrum of compound **8** (400 MHz, CDCl_3_)

**Figure S37.** ^13^C NMR spectrum of compound **8** (101 MHz, CDCl_3_)

**Figure S38.** ^1^H NMR spectrum of compound **9** (400 MHz, CDCl_3_)

**Figure S39.** ^13^C NMR spectrum of compound **9** (101 MHz, CDCl_3_)

**Figure S40.** ^13^C DEPT spectrum of compound **9** (101 MHz, CDCl_3_)

**Figure S41.** ^1^H NMR spectrum of compound **11** (400 MHz, CDCl_3_)

**Figure S42.** ^13^C NMR spectrum of compound **11** (101 MHz, CDCl_3_)

**Figure S43.** ^1^H NMR spectrum of compound **1** (400 MHz, CDCl_3_)

**Figure S44.** ^13^C NMR spectrum of compound **1** (101 MHz, CDCl_3_)

**Figure S45.** ^13^C DEPT spectrum of compound **1** (101 MHz, CDCl_3_)

**Figure S46.** ^1^H NMR spectrum of compound **13** (400 MHz, CDCl_3_)

**Figure S47.** ^13^C NMR spectrum of compound **13** (101 MHz, CDCl_3_)

**Figure S48.** ^1^H NMR spectrum of compound **14** (400 MHz, CDCl_3_)

**Figure S49.** ^13^C NMR spectrum of compound **14** (101 MHz, CDCl_3_)

**Figure S50.** ^1^H NMR spectrum of compound **2** (400 MHz, DMSO-*d*_6_)

**Figure S51.** ^13^C NMR spectrum of compound **2** (101 MHz, DMSO-*d*_6_)

**Figure S52.** ^13^C DEPT spectrum of compound **2** (101 MHz, DMSO-*d_6_*)

**Figure S53.** ^1^H NMR spectrum of compound **15** (400 MHz, CDCl_3_)

**Figure S54.** ^13^C NMR spectrum of compound **15** (101 MHz, CDCl_3_)

**Figure S55.** ^13^C DEPT spectrum of compound **15** (101 MHz, CDCl_3_)

**Figure S56.** ^1^H NMR spectrum of compound **16** (400 MHz, CDCl_3_)

**Figure S57.** ^13^C NMR spectrum of compound **16** (101 MHz, CDCl_3_)

**Figure S58.** ^13^C DEPT spectrum of compound **16** (101 MHz, CDCl_3_)

**Figure S59.** ^1^H NMR spectrum of compound **17** (400 MHz, CD_3_OD)

**Figure S60.** ^13^C NMR spectrum of compound **17** (101 MHz, CD_3_OD)

**Figure S61****.** ^13^C DEPT spectrum of compound **17** (101 MHz, CD_3_OD)

**Figure S62.** ^1^H NMR spectrum of SBA-C (400 MHz, CDCl_3_)

**Figure S63.** ^13^C NMR spectrum of SBA-C (101 MHz, CDCl_3_)

**Figure S64.** ^13^C DEPT spectrum of SBA-C (101 MHz, CDCl_3_)

**Table S1.** Comparison of the ¹H NMR data of the synthetic and the natural SBA-C (400 MHz, CDCl_3_)

| No. | Assignment | Natural Product δ  (ppm, mult, *J* in Hz)* | Synthetic Product δ  (ppm, mult, *J* in Hz) | Δδ  （ppm） |
| --- | --- | --- | --- | --- |
| 1 | 2′‑H | 6.78 (d, *J* = 2.0, 1H) | 6.78 (d, *J* = 2.0, 1H) | 0 |
| 2 | 5′‑H | 6.70 (d, *J* = 8.0, 1H) | 6.70 (d, *J* = 8.0, 1H) | 0 |
| 3 | 5''''‑H | 6.68 (d, *J* = 8.0, 1H) | 6.68 (d, *J* = 8.0, 1H) | 0 |
| 4 | 5″‑H | 6.67 (d, *J* = 8.0, 1H) | 6.67 (d, *J* = 8.0, 1H) | 0 |
| 5 | 2″‑H | 6.66 (d, *J* = 2.0, 1H) | 6.66 (d, *J* = 2.0, 1H) | 0 |
| 6 | 2‴‑H | 6.66 (d, *J* = 2.0, 1H) | 6.66 (d, *J* = 2.0, 1H) | 0 |
| 7 | 5‴‑H | 6.64 (d, *J* = 8.0, 1H) | 6.64 (d, *J* = 8.0, 1H) | 0 |
| 8 | 2''''‑H | 6.63 (d, *J* = 2.0, 1H) | 6.63 (d, *J* = 2.0, 1H) | 0 |
| 9 | 6′‑H | 6.53 (dd, *J* = 8.0, 2.0, 1H) | 6.53 (dd, *J* = 8.0, 2.0, 1H) | 0 |
| 10 | 6″‑H | 6.48 (dd, *J* = 8.0, 2.0, 1H) | 6.48 (dd, *J* = 8.0, 2.0, 1H) | 0 |
| 11 | 6‴‑H | 6.45 (dd, *J* = 8.0, 2.0, 1H) | 6.45 (dd, *J* = 8.0, 2.0, 1H) | 0 |
| 12 | 6''''‑H | 6.35 (dd, *J* = 8.0, 2.0, 1H) | 6.35 (dd, *J* = 8.0, 2.0, 1H) | 0 |
| 13 | α-H | 4.73 (dd, *J* = 7.5, 5.0, 1H) | 4.72 (dd, *J* = 7.3, 5.2, 1H) | 0.01 |
| 14 | α'-H | 4.59 (dd, *J* = 6.0, 6.0, 1H) | 4.57 (t, *J* = 5.9, 1H) | 0.02 |
| 15 | C-1-H, C-3-H | 4.21 (brdd, *J* = 10.5, 7.0, 2H) | 4.25–4.14 (m, 2H) | Not resolved |
| 16 | β-H_α_ | 3.91 (dd, *J* = 10.5, 7.0, 1H) | 3.91 (dd, *J* = 10.5, 7.5, 1H) | 0 |
| 17 | β'-H_β_ | 3.75 (dd, *J* = 10.5, 7.0, 1H) | 3.75 (dd, *J* = 10.2, 6.8, 1H) | 0 |
| 18 | β-H_1_ | 2.86 (dd, *J* = 14.0, 5.0, 1H) | 2.87 (dd, *J* = 14.1, 5.1, 1H) | 0.01 |
| 19 | β-H_2_ | 2.81 (dd, *J* = 14.0, 7.5, 1H) | 2.81 (dd, *J* = 14.1, 7.7, 1H) | 0 |
| 20 | β'-H_1_ | 2.71 (dd, *J* = 14.0, 6.0, 1H) | 2.72 (dd, *J* = 14.1, 6.2, 1H) | -0.01 |
| 21 | β'-H_2_ | 2.61 (dd, *J* = 14.0, 6.0, 1H) | 2.60 (dd, *J* = 14.1, 5.7, 1H) | 0.01 |

**Table S2.** Comparison of the ^13^C NMR data of the synthetic and the natural SBA-C (101 MHz, CDCl_3_)

| No. | Assignment | Natural Product δ  (ppm) * | Synthetic Product δ  (ppm) | Synthetic Product  δ (ppm) rounded | Δδ  (ppm) |
| --- | --- | --- | --- | --- | --- |
| 1 | COOH‑1 | 173.4 | 173.37 | 173.4 | 0 |
| 2 | COOH‑2 | 173.2 | 173.29 | 173.3 | 0 |
| 3 | Ester‑C=O‑1 | 172.8 | 172.83 | 172.8 | 0 |
| 4 | Ester‑C=O‑2 | 172.7 | 172.70 | 172.7 | 0 |
| 5 | C‑3′ | 146.2 | 146.15 | 146.2 | 0 |
| 6 | C‑4′ | 146.1 | 146.09 | 146.1 | 0 |
| 7 | C‑3″ | 146.0 | 145.93 | 145.9 | 0.1 |
| 8 | C‑4″ | 145.9 | 145.87 | 145.9 | 0 |
| 9 | C‑3‴ | 145.5 | 145.40 | 145.4 | 0.1 |
| 10 | C‑4‴ | 145.3 | 145.26 | 145.3 | 0 |
| 11 | C‑3'''' | 145.2 | 145.16 | 145.2 | 0 |
| 12 | C‑4'''' | 145.2 | 145.11 | 145.1 | 0.1 |
| 13 | C‑1′ | 131.7 | 131.62 | 131.6 | 0.1 |
| 14 | C‑1″ | 131.6 | 131.50 | 131.5 | 0.1 |
| 15 | C‑1‴ | 128.9 | 128.83 | 128.8 | 0.1 |
| 16 | C‑1'''' | 128.6 | 128.53 | 128.5 | 0.1 |
| 17 | C‑6′ | 122.2 | 122.18 | 122.2 | 0 |
| 18 | C‑6″ | 122.2 | 122.15 | 122.2 | 0 |
| 19 | C‑6‴ | 120.5 | 120.51 | 120.5 | 0 |
| 20 | C‑6'''' | 119.5 | 119.34 | 119.3 | 0.2 |
| 21 | C‑2′ | 117.8 | 117.77 | 117.8 | 0 |
| 22 | C‑2″ | 117.6 | 117.49 | 117.5 | 0.1 |
| 23 | C‑5′ | 116.5 | 116.46 | 116.5 | 0 |
| 24 | C‑5″ | 116.4 | 116.399 | 116.4 | 0 |
| 25 | C‑5‴ | 116.4 | 116.398 | 116.4 | 0 |
| 26 | C‑2‴ | 116.3 | 116.24 | 116.2 | 0.1 |
| 27 | C‑2'''' | 116.0 | 115.98 | 116.0 | 0 |
| 28 | C‑5'''' | 115.6 | 115.49 | 115.5 | 0.1 |
| 29 | α‑C | 74.9 | 74.91 | 74.9 | 0 |
| 30 | α′‑C | 74.9 | 74.81 | 74.8 | 0.1 |
| 31 | Cyclobutane C‑1 | 48.7 | 48.57 | 48.6 | 0.1 |
| 32 | Cyclobutane C‑3 | 48.0 | 47.88 | 47.9 | 0.1 |
| 33 | β‑CH₂ | 42.8 | 42.74 | 42.7 | 0.1 |
| 34 | β′‑CH₂ | 42.1 | 41.99 | 42.0 | 0.1 |
| 35 | Cyclobutane C‑2 | 37.9 | 37.80 | 37.8 | 0.1 |
| 36 | Cyclobutane C‑4 | 37.6 | 37.48 | 37.5 | 0.1 |

Note ：* data from the previous report. Murata T., Miyase T., Yoshizaki F. Hyaluronidase Inhibitors from Keiskea Japonica [J]. *Chemical and Pharmaceutical Bulletin*, 2012, *60*(1): 121-128.

The experimental setup of the small-scale synthesis of compound **2**

**Figure S65.** Reconstruction of the small-scale experimental setup

Information of the black-light lamp: brand Xuelaite (Cnlight), Product Model 3U-40w, operating voltage 220 volts, E27 socket, inside coated with phosphor, peak wavelength 365 nm. The photodimerization experiments were carried out in a fume hood. The door of the fume hood was completely covered with aluminum foils and was kept closed during the experiments.

To a 25 ml pear-shaped Pyrex^®^ glass flask, added powder of compound **14** (0.11 g, 0.5 mmol), EtOH/H_2_O (5:95) and a magnetic stirring bar. The flask was sealed with a rubber stopper. The heterogenous mixture was magnetically stirred to form a relatively uniform suspension. A black-light lamp was put about 2 cm away from the reaction flask. The reaction progressed was monitored by HPLC analysis. After completion, usually 24-48 h, the stirrer was stopped and the flask was taken out. The off-white suspension was filtered and the solid was recrystallized from absolute ethanol to afford compound **2** as white crystals.

Large-scale and improved synthesis of compound **2**

**Figure S66.** Snapshot and devices of the large-scale experimental setup

Information of the LED UV light lamp: brand Yixian, size 180 x 140 x 105 mm, operating voltage 220 volts, power 30 W, peak wavelength 365 nm, UV irradiance 86 mw/cm^2^. The photodimerization experiments were carried out in a fume hood. The door of the fume hood was completely covered with aluminum foils and was kept closed during the experiments.

To a 250 mL Pyrex^®^ glass flask, was added compound **14** (2 g, 9 mmol), 100 mL water and two irregular Teflon bars. The flak was sealed with a serrated septum and fastened to a steel bottle-grabber with a nylon cable tie. The grabber was installed onto an overhead mechanical stirrer and fastened tightly. The mechanical stirrer was set to rotate constantly at 100-150 rpm. To prevent the bottle-grabber from wobbling during long-time working, a flask-holder was equipped just above the grabber to limit its position. The LED UV-light lamp was put under the rotating flask tilted about 45 degrees toward the inside of the hood. The flask was kept at a distance of 2-4 cm to the lamp, to ensure enough exposure to UV irradiation and to avoid possible collisions.

The reaction progressed was monitored by HPLC analysis. After completion, usually 24-48 h, the stirrer was stopped and the flask was taken out. The off-white suspension was filtered and the solid was recrystallized from absolute ethanol to afford compound **2** as white crystals.

X-ray crystallographic data of compound **14**

Compound **14** was dissolved in ethyl acetate and then diluted with hexane. The solution was placed in a refrigerator, and colorless, grain-like crystals slowly formed. X‑ray diffraction analysis was performed by Zhongke Baice (<https://www.zkbaice.cn/>). The diffraction data show that the olefinic double bond of **14** is disordered and can adopt two zigzag conformations (Figure S67): conformation A (30%) and conformation B (70%). This disorder does not affect the head‑to‑tail photodimerization, and the crystal structure of **14** is illustrated using only conformation B.

**Figure S67.** Single crystal XRD spectrum of compound **14**

X-ray Crystallographic Data of Compound **2**

**Figure S68.** Single crystal XRD spectrum of compound **2**
